# SNP-based genetic linkage map of tobacco (*Nicotiana tabacum* L.) using next-generation RAD sequencing

**DOI:** 10.1186/s40709-015-0034-3

**Published:** 2015-10-06

**Authors:** Bingguang Xiao, Yuntao Tan, Ni Long, Xuejun Chen, Zhijun Tong, Yang Dong, Yongping Li

**Affiliations:** Yunnan Academy of Tobacco Agricultural Science, Yuantong Street No. 33, Kunming, 650021 Yunnan China; Faculty of Life Science and Technology, Kunming University of Science and Technology, JingMing South Road No. 727, Kunming, 650500 Yunnan China

**Keywords:** Tobacco, Linkage map, *Nicotiana tabacum* L., RAD sequencing, SNP

## Abstract

**Background:**

Tobacco (*Nicotiana tabacum* L.) is an important model system, which has been widely used in plant physiological studies and it is particularly useful as a bioreactor. Despite its importance, only limited molecular marker resources are available for genome analysis, genetic mapping and breeding. Restriction-site associated DNA sequencing (RAD-seq) is a powerful new method for targeted sequencing across the genomes of many individuals. This approach has broad potential for genetic analysis through linkage mapping.

**Results:**

We constructed a RAD library using genomic DNA from a BC_1_ backcross population. Sequencing of 196 individuals was performed on an Illumina HiSeq 2500. Two linkage maps were constructed, one with a reference genome and another, termed as de novo identification of single nucleotide polymorphism (SNP) by RAD-seq, without a reference genome. Overall, 4138 and 2162 SNP markers with a total length of 1944.74 and 2000.9 cM were mapped to 24 linkage groups in the genetic maps based on reference genome and without reference, respectively.

**Conclusions:**

Using two different SNP discovery methods based on next generation RAD sequencing technology, we have respectively mapped 2162 and 4318 SNPs in our backcross population. This study gives an excellent example for high density linkage map construction, irrespective of genome sequence availability, and provides saturated information for downstream genetic investigations such as quantitative trait locus analyses or genomic selection (e.g. bioreactor suitable cultivars).

**Electronic supplementary material:**

The online version of this article (doi:10.1186/s40709-015-0034-3) contains supplementary material, which is available to authorized users.

## Background

Tobacco (*Nicotiana tabacum* L., 2n = 4x = 48) is an important model system in plant biotechnology [1], due to its unique advantages over other plant species. It not only has relatively short generation time and high protein content, but also can be easily genetically transformed [2, 3]. For this reason, tobacco has been widely used in studies on plant response to pathogens [4], pyridine alkaloid (like nicotine) biosynthesis [5], cell cycle [6, 7], oxidative stress [8] and pollen tube development [9]. More importantly, tobacco is an attractive green bioreactor proved to be able to produce a wide range of therapeutic proteins including antibodies [10–12], vaccines [13, 14] and immunomodulatory molecules such as cytokines [15, 16].

Despite the prospective applications of tobacco in pharmaceutical production, limited cultivars exist with low nicotine and alkaloid contents. Breeding new cultivars suitable for pharmaceutical production is further complicated by the paltry genomic information available to the public. Genetic linkage mapping based on molecular markers permits the elucidation of genome structure and organization [17]. It provides critical information for quantitative trait locus (QTL) marker assisted selection. For some economic plants, including potato (*Solanum tuberosum*), tomato (*Solanum lycopersicum*), eggplant (*Solanum melongena*), pepper (*Capsicum species*) and Petunia (*Petunia hybrida*), whole genome sequencing and genetic linkage maps have elucidated their genome structures and assisted breeding cultivars with molecular markers [18]. Therefore, a high density genome-based linkage map of the tetraploid tobacco will improve current genetic research tools in search of new cultivars. Thus far, linkage maps for tobacco have been constructed by using low-throughput molecular markers like simple sequence repeats (SSRs), which resulted in low density linkage maps [19, 20].

Single nucleotide polymorphisms (SNPs) as the most abundant type of DNA variations are currently used as genetic markers for their wide distribution in the genome [21]. Compared to genetic markers based on size discrimination or hybridization, SNPs directly interrogate sequence variation and possess the potential of reducing genotyping errors [22]. SNP discovery is amenable to high-throughput next-generation sequencing (NGS) technologies, which produce DNA sequences at a rate several orders of magnitude faster than conventional sequencing methods [17].

According to unpublished data, the genome size of tobacco is approximately 4.5 Gb. Because of the huge genome, great challenges must be faced up to. Reduced representation library sequencing is an energetic approach, which has been used for many genome studies [23]. Restriction site associated DNA sequencing (RAD-seq) technology [24–26] facilitates genetic variant discovery by allowing ortholog sequences to be targeted in multiple individuals [27]. This method relies on sequencing of DNA regions flanking the restriction sites of specific restriction enzymes. In brief, DNA fragments from the digestion of a chosen restriction enzyme are ligated with an adapter, which contains a molecular identifying sequence (MID) unique to each sample. The DNA sequences flanking each restriction site are sequenced via the massively parallel Illumina sequencing technology [28]. RAD sequencing is highly successful in re-identifying genomic regions controlling known phenotypes [29–31].

To generate a high density genome linkage map for tobacco, we have developed here 4138 SNP markers using the Illumina HiSeq 2500 high-throughput platform. The mapping population was generated by crossing two tobacco (*N. tabacum* L.) cultivars. The F_1_ progeny was back-crossed to the parents. A total of 193 progenies were generated and all individuals were used for linkage map construction. We conducted SNP detection both with and without a reference genome, the latter referred to as de novo identification of SNP by RAD-seq (DISR). We compared these two methods and constructed a genetic map of tobacco based on a backcross (BC_1_) population.

## Results

### RAD library preparation and sequencing

**Fig. 1:**
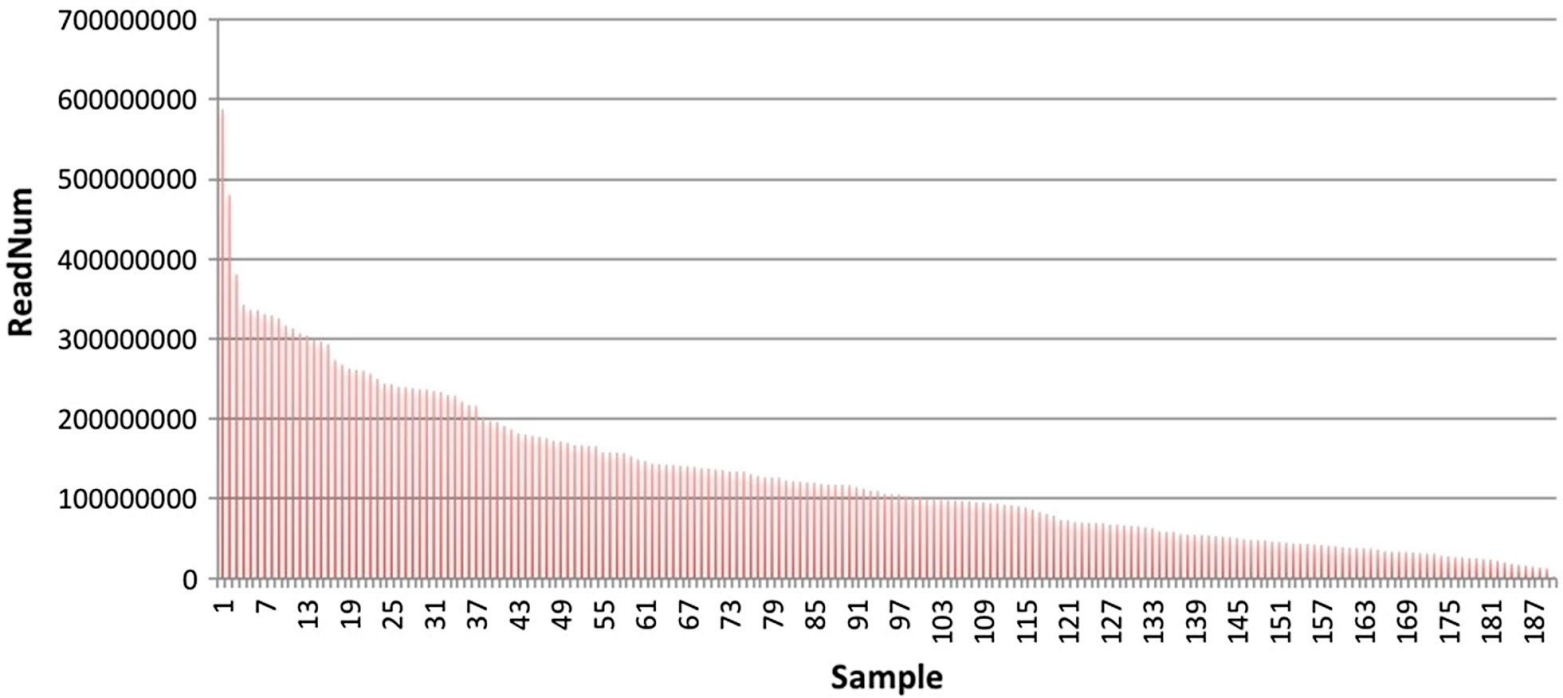
The statistic of read number for each sample

A total of 196 sampled individuals from three generations, HD (Hong hua Da jin yuan), RBST (Resistance to Black Shank Tobacco), F_1_ (HD × RBST) and 193 BC_1_ progenies were used in the construction of 10 libraries used for RAD-sequencing (Table 1). In summary, 2641 Gb of raw data containing 26.4 billion pair-end 2 × 100 bp raw reads for approximately 2640 billion base pairs were obtained. Library detail information is provided in Additional file 1. We removed the following types of reads: (a) reads with >10 % unidentified nucleotides (N), (b) reads with >40 bases having Phred quality ≤7, and (c) putative PCR duplicates generated by PCR amplification in the library construction process (i.e., read 1 and read 2 of two paired-end reads that were completely identical). These reads were stringently filtered from the index sequences to get clean data for each sample (Fig. 1). Totally, 2481 Gb clean data contain 24.8 billion clean reads after filtering with an average volume of 12.11 Gb for each sample, at an average sequencing depth of 2.7× (the unpublished tobacco genome size is approximately 4.5 Gb).

**Table 1:** Library information and data output

Lib	Raw reads (M)	Raw bases (Gb)	Clean reads (M)	Clean bases (Gb)	GC rate (%)	Q20 rate (%)
1	2251.27	225.13	2173.69	217.37	42.25	97.54
2	2612.37	261.24	2322.90	232.29	40.56	97.15
3	2622.27	262.23	2497.09	249.71	40.49	96.44
4	2456.33	245.63	1931.97	193.20	40.71	95.93
5	2765.69	276.57	2658.79	265.88	41.37	96.35
6	2750.33	275.03	2658.93	265.89	40.48	96.46
7	2410.54	241.05	2334.68	233.47	40.59	97.20
8	2867.82	286.78	2757.00	275.70	41.18	96.53
9	2912.57	291.26	2802.38	280.24	40.86	95.85
10	2761.11	276.11	2673.15	267.31	40.96	96.93
Total	26,410.31	2641.03	24,810.56	2481.06	40.95	96.64

Basic statistical information about the RAD sequencing is presented, which contain the raw reads, raw bases, clean reads and clean base after filtered, GC rate mean value and Q20 mean value

### SNP calling and genotyping

Two distinct protocols were executed in SNP calling and genotyping: the first was with a reference genome; the second was without a reference genome, which we refer to as DISR. In the first protocol, 24.8 billion clean reads were aligned to the reference sequences (unpublished data) using SOAPaligner [32] (Release 2.21, http://soap.genomics.org.cn/). The mapping results were processed with Samtools [33]. Variations were called using the Unified Genotyper (Version 3.1, Genome Analysis Tool Kit) [34]. Any nucleotide difference between reads and the reference genome was initially called as variant. A large volume output of 7,343,419 raw SNPs suggested improvement in data assemblage. Three parameters (genotype coverage, genotype quality, and SNP quality) generated by the Unified Genotyper were used as criteria for filtering variant output.

Using a maximum missing data (MMD) threshold of 45 % in the BC_1_ population for each locus, a total of 8664 SNPs (*p* < 0.01) were recovered. Although the criteria are much looser than many other studies [31], the effective genotype size is larger than 100, which is sufficient for linkage map construction. In total, 5286 markers (*χ*^2^ < 15) were selected for genetic map construction by using JoinMap 4.0 [35] (Table 2).

**Table 2:** Statistics for SNPs based on the two different methods

Method of SNPcalling	Raw SNPs	Clean SNPs	*χ* ^2^ < 15
DISR^a^	181,770	7457	3282
Based on reference genome	7,343,419	8664	5286

Shown are the number of raw SNPs, the number of SNPs remained after filtering and the number of SNPs by a Chi square test

^a^De novo identification of SNP by RAD-seq

In the second protocol (DISR), 181,770 raw SNPs were obtained after the clean reads were processed. Using the same MMD threshold as the first protocol, a total of 7457 SNPs (*p* < 0.01) were recovered. In total, 3282 markers were then selected (by the *χ*^2^ test) for the construction of genetic map in JoinMap 4.0 [35] (Table 2).

### Linkage mapping

**Fig. 2:**
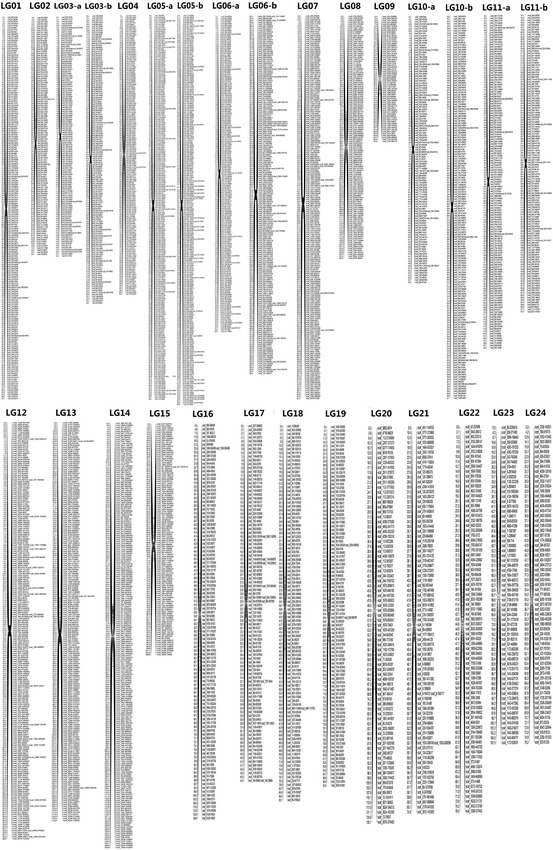
Linkage maps based on the reference genome. This was constructed with a total of 8664 SNPs (*p* < 0.01) which generated 4138 markers mapping 24 linkage groups (LGs) successfully with a total length of 1944.74 cM. The LGs distance ranged from 33.58 to 129.176 cM. Six LGs contained over 220 marker loci and for these LGs Haldane’s map unit is used while for other LGs we used Kosambi’s map unit. The LG09, LG23 and LG24 were the shortest LGs, spanning 73.937–107.485 cM, respectively, and comprising 65 loci, whereas LG05 was the longest LG, spanning 60.73 cM and containing 494 loci with a marker density of 0.123 cM/locus. The marker densities ranged from 0.117 cM/locus in LG12 to 1.679 cM/locus in LG23, resulting in an average distance of 0.712 cM between markers for the entire map

The first linkage map from sequence with reference genome was constructed with a total of 8664 SNPs (*p* < 0.01) which generated 4138 markers and mapped 24 linkage groups (LGs) successfully with a total length of 1944.74 cM. The LGs ranged from 33.58 to 129.176 cM in length. Six LGs contained over 220 marker loci. LG09, LG23 and LG24 were the shortest LGs, spanning 73.937–107.485 cM, respectively, and comprising 65 loci, whereas LG05 was the largest LG, spanning 60.73 cM, containing 494 loci with marker density of 0.123 cM/locus. The marker densities ranged from 0.117 cM/locus in LG12 to 1.679 cM/locus in LG23, resulting in an average distance of 0.712 cM between markers for the entire map (Table 3; Fig. 2).

**Table 3:** Statistics of 24 linkage groups with the reference genome

Linkage group	Marker no.	Size (cM)	Marker density (cM/loci)
LG01	190	67.024	0.355
LG02	116	76.268	0.663
LG03	280	33.580	0.120
LG04	161	105.310	0.658
LG05	494	60.780	0.123
LG06	402	50.368	0.126
LG07	206	52.656	0.257
LG08	125	129.176	1.042
LG09	65	73.937	1.155
LG10	294	37.134	0.127
LG11	279	37.254	0.134
LG12	227	26.454	0.117
LG13	199	115.937	0.586
LG14	209	150.580	0.724
LG15	115	106.013	0.930
LG16	105	126.780	1.219
LG17	103	47.001	0.461
LG18	100	93.132	0.941
LG19	97	90.641	0.944
LG20	81	130.057	1.626
LG21	81	74.543	0.932
LG22	79	76.465	0.980
LG23	65	107.485	1.679
LG24	65	76.166	1.190
Total	4138	1944.741	0.712

Shown are the marker number, linkage size (cM), and marker density of each linkage group. LG5 contains a maximum marker number of 494. The LGs distance ranged from 33.58 to 129.176 cM. Six LGs contained over 220 marker loci. For these LGs Haldane’s map unit is used while for other LGs we used Kosambi’s map unit

**Fig. 3:**
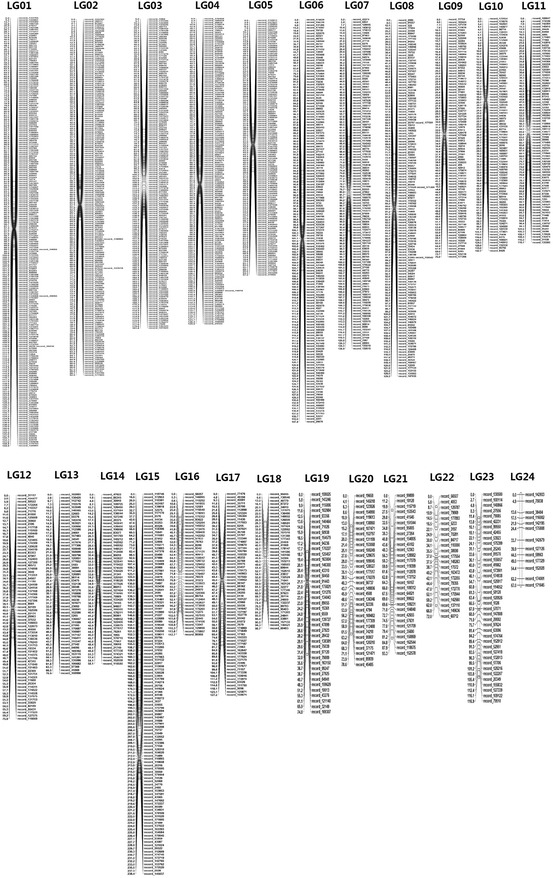
Linkage maps based on DISR. This map was constructed with 7457 SNPs that produced 3282 markers. Out of those, 2162 markers successfully mapped 24 LGs with a total length of 2700.9 cM. The LGs ranged from 58.1 to 238.4 cM in length. LG24 was the shortest LG, comprising only 13 loci, whereas LG01 was the longest, spanning 159.9 cM and containing 224 loci with a marker density of 0.7 cM/locus (map unit determined by Haldane’s distance while for other LGs Kosambi’s distance was used). The marker densities ranged from 0.5 cM/locus in LG02 to 5.6 cM/locus in LG24, resulting in an average distance of 1.8 cM between markers for the entire map

The second linkage map from DISR was constructed with 7457 SNPs that gave 3282 markers. Out of those, 2162 markers successfully mapped 24 LGs with a total length of 2700.9 cM. The LGs ranged from 58.1 to 238.4 cM in length, and only one LG contained over 220 marker loci. LG24 was the shortest LG, comprising only 13 loci, whereas LG01 was the largest LG, spanning 159.9 cM and containing 224 loci with marker density of 0.7 cM/locus. The marker densities ranged from 0.5 cM/locus in LG02 to 5.6 cM/locus in LG24, resulting in an average distance of 1.8 cM between markers for the entire map (Table 4; Fig. 3).

**Table 4:** Statistics of 24 linkage groups without the reference genome (DISR)

Linkage group	Marker no.	Size (cM)	Marker density (cM/loci)
LG01	224	159.9	0.7
LG02	186	92.5	0.5
LG03	160	181.8	1.1
LG04	159	129.6	0.8
LG05	132	97.2	0.7
LG06	150	147.4	1.0
LG07	123	138.8	1.1
LG08	136	129.5	1.0
LG09	89	75.9	0.9
LG10	87	142.7	1.7
LG11	84	116.1	1.4
LG12	71	75.9	1.1
LG13	57	70.9	1.3
LG14	54	58.1	1.1
LG15	120	238.4	2.0
LG16	45	117.7	2.7
LG17	64	127.2	2.0
LG18	43	98.7	2.4
LG19	41	74.8	1.9
LG20	32	78.6	2.5
LG21	30	93.3	3.2
LG22	23	72.0	3.3
LG23	39	116.9	3.1
LG24	13	67.0	5.6
Total	2162	2700.9	1.8

Shown are the marker number, linkage size (cM), and marker density of each linkage group. LG24 was the shortest LG, comprising only 13 loci, whereas LG01 was the longest, spanning 159.9 cM and containing 224 loci with a marker density of 0.7 cM/locus (map unit determined by Haldane’s distance while for other LGs Kosambi’s was used)

### Comparison of the DISR and the reference genome methods

**Fig. 4:**
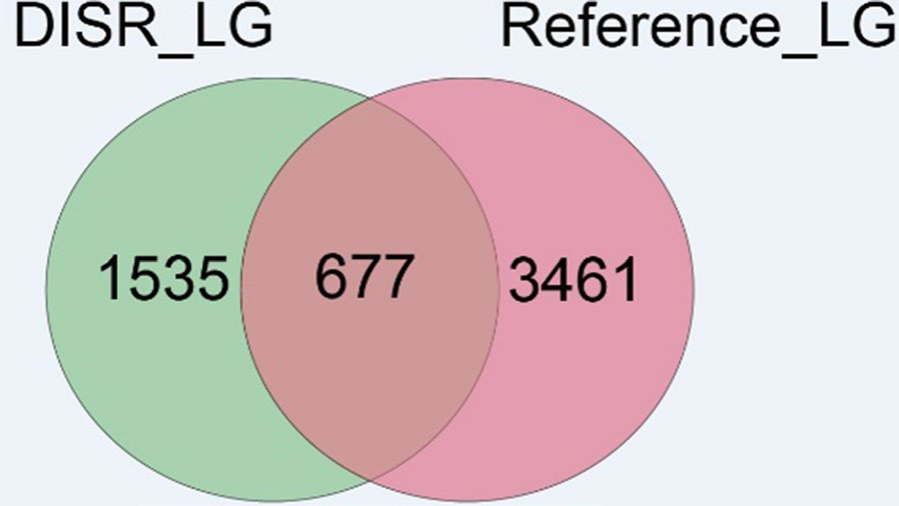
Comparison of the two map versions. In total, 677 overlapping markers, constituting 30 % of the DISR map and 16 % of the map based on the reference genome were observed. All in all, 1535 makers were specified for the DISR map and 3461 markers for the map based on the reference genome

Comparison was performed by presenting the ratio of the marker overlaps between the genetic maps based on reference genome and DISR. The consensus sequence was mapped back to the reference genome to mark the loci of the SNPs. After this process, the markers from the DISR method were compared with the markers generated from the reference genome method. Consistent markers were recorded and presented as a Venn diagram. In total, 677 overlapping markers, constituting 30 % of the DISR map and 16 % of the map based on reference genome were observed. All in all, 1535 makers were specified for the DISR map and 3461 markers for the map based on reference genome (Fig. 4).

## Discussion

Although tobacco has been proved to be an attractive green bioreactor for the production of therapeutic proteins, the paucity of cultivars with low nicotine and alkaloid contents has blocked its movement from bench to field scale. A high density genetic map can provide sufficient information to accelerate the genome breeding. Previous attempts for genetic linkage map construction for tobacco were achieved by using molecular marker based techniques, including restriction fragment length polymorphism (RFLP) [36], conserved ortholog sequences (COS) [37] and simple sequence repeat (SSR) markers [19, 20]. As the best of the three linkage maps, the SSR linkage map comprises 2318 SSR markers mapping to 2363 loci in 24 clearly defined LGs with a total length of 3270 cM [19] (Table 5). In comparison, our technique generated 4138 SNP markers for tobacco that defined 24 LGs with a total coverage of 1944.7 cM. This result is not only an improvement over those of previous reports, but also a confirmation of SNPs in providing excellent marker density for linkage mapping and genomic selection [38]. To our knowledge, the tobacco linkage maps from this study, particularly the map generated with a reference genome, provide the highest number of markers among all available population-specific linkage maps.

**Table 5:** Comparison of linkage maps for tobacco

Cross combination	Population type	Type of markers	No. of markers	Map length (cM)	Groups	References
Hicks Broadleaf × Red Russian	F_2_	SSR^a^	286	1920	26	[20]
Flue-cured tobacco Taiyan 7 × burley tobacco cultivar Bailei 21	F_2_	SRAP^b^/ISSR^c^	112	1560.2	26	[47]
Burley37 × Burley21	DH^d^	SRAP/AFLP^e^	118	1953.6	22	[48]
Hicks Broadleaf × Red Russian	F_2_	SSR	2317	3270	24	[19]
Hicks Broadleaf × HHDJY(HD)	DH	SSR/DArT^f^	851	2291	24	[49]
Honghua Dajinyuan (HD) × HBL	DH	SSR	611	1882.1	24	[50]
(HD × RBST) × HD	BC_1_	SNP	4138	1944.7	24	This study
(HD × RBST) × HD	BC_1_	SNP	2162	2700.9	24	This study

^a^Simple sequence repeats

^b^Sequence related amplified polymorphism

^c^Inter-simple sequence repeat

^d^Doubled haploid population

^e^Amplified fragment length polymorphism

^f^Diversity arrays technology

The Mendelian basis of quantitative traits provides a genetic framework for the dissection of polygenic traits [39] and can pave the way for the identification of candidate loci controlling the inheritance of complex traits. NGS technology makes it possible to achieve dense SNP marker coverage of genomes without the need for a reference sequence [24, 26]. An example of this is restriction-associated DNA sequencing (RAD-seq), which was originally developed as a tool for genetic mapping in fish and fungi [29] and later expanded to many other species, including plants (*Lolium perenne* L., *Momordica charantia*, *Corchorus olitorius* L.) [25, 30, 40, 41]. In this study, a separate linkage map via the DISR method was also obtained, which did not need a reference genome. The DISR linkage map contains 2162 markers with a total coverage of 2700.9 cM and an average distance of 1.8 cM between markers. It demonstrates that these two high density linkage maps are compelling tools for gene (Table 5) and QTL mapping and marker-assisted breeding [42].

A comparison of the two maps showed an overlap of 677 markers (Fig. 4). We compared the ratios of overlaps between the two protocols and found that the use of a reference genome was more efficient than without a reference genome. In the method of DISR, the information of only one end of the pair reads is used for the SNP calling. However, if we conduct the SNP calling with a reference genome, whole genome information is used. This kind of experiment is often required in nature, particularly in building linkage maps for species that do not have a complete genome sequence database. However, an integration of the two protocols could result in a higher density map and thus, assist in the breeding of other low nicotine and alkaloid content cultivars.

## Conclusions

Using next generation RAD sequencing technology for two distinct SNP discovery methods, we have respectively mapped 2162 and 4318 SNPs in tobacco. This study gives an excellent example for high density linkage map construction, irrespective of reference genome sequence availability, and provides saturated information for downstream genetic investigations such as QTL analyses or genomic selection (e.g. bioreactor suitable cultivars).

## Methods

### Mapping population

Two tobacco varieties, Hong hua Da jin yuan (HD) and Resistance to Black Shank Tobacco (RBST) were used to develop the BC_1_ inbred population. HD is a high leaf mass cultivar from southwest of China. RBST has high resistance to tobacco black shank disease. The BC_1_ inbred population was generated through a (HD × RBST) × HD crossing in a breeding unit in Yuxi of Yunnan Province.

### RAD library preparation and sequencing

Fresh young leaves were collected from HD, RBST, F_1_ (HD × RBST) and 193 individuals of BC_1_ (F_1_ × HD) population. Leaf samples were snap frozen in liquid nitrogen and stored at −80 °C. Genomic DNA isolation and purification were conducted using a DNA extraction kit (Qiagen). DNA quality was analyzed in 1 % agarose gel. The concentration of extracted DNA was determined by a spectrophotometer. Approximately 15 μg of purified DNA was processed to obtain 10 RAD libraries, each including about 20 individuals following the protocol of Baird et al. [29] and the instructions of the reagent manufacturers. Genomic DNA from individual samples was digested with *Eco*RI (New England Biolabs). Individual specific barcodes were ligated with an adaptor by T4 DNA ligase for sample multiplexing. Ligated DNA samples were pooled and sheared, and consequently electrophoresed to isolate DNA fragments with sizes of 300–700 bp in 1.5 % agarose gel. Quick Blunting Kit (New England Biolabs) was used to generate phosphorylated blunt ends. Klenow Fragment (3′ → 5′ exo-; New England Biolabs) was used to add adenosine to the 3′ end. An adapter with divergent ends (P2 adapter) was ligated to enable selective PCR. The samples were PCR-amplified and the libraries purified with MinElute column (Qiagen) to obtain approximately 100 μl (>50 ng μl^−1^) of sequencing libraries. The obtained RAD libraries were sequenced on an Illumina HiSeq 2500 in 100 bp pair-end reads.

### SNP calling with reference genome

The raw reads were removed using the following criteria: (a) reads with >10 % unidentified nucleotides (N), (b) reads with >40 bases having Phred quality ≤7, and (c) putative PCR duplicates generated by PCR amplification in the library construction process (i.e., read 1 and read 2 of two paired-end reads that were completely identical). All the obtained short clean reads were aligned to reference sequences (unpublished data) using SOAPaligner (Release 2.21, http://soap.genomics.org.cn/) [32]. During alignment, long reads with high error rates at 3′-ends were substituted with 5′ 32 bp subsequence as seeds. The entire lengths of the reads were used. Five mismatches in one read were allowed (important arguments: -l 32 -v 5). The mapping results SAM files were converted with Samtools [33]. Variations were called using the Unified Genotyper (Version 3.1, Genome Analysis Tool Kit) [34]. Any nucleotide difference between reads and the reference genome was identified as a variant. This criterion generated a large variant output, which was filtered by three parameters generated with the Unified Genotyper, including genotype coverage, genotype quality, and SNP quality.

### SNP calling without reference genome (DISR)

**Fig. 5:**
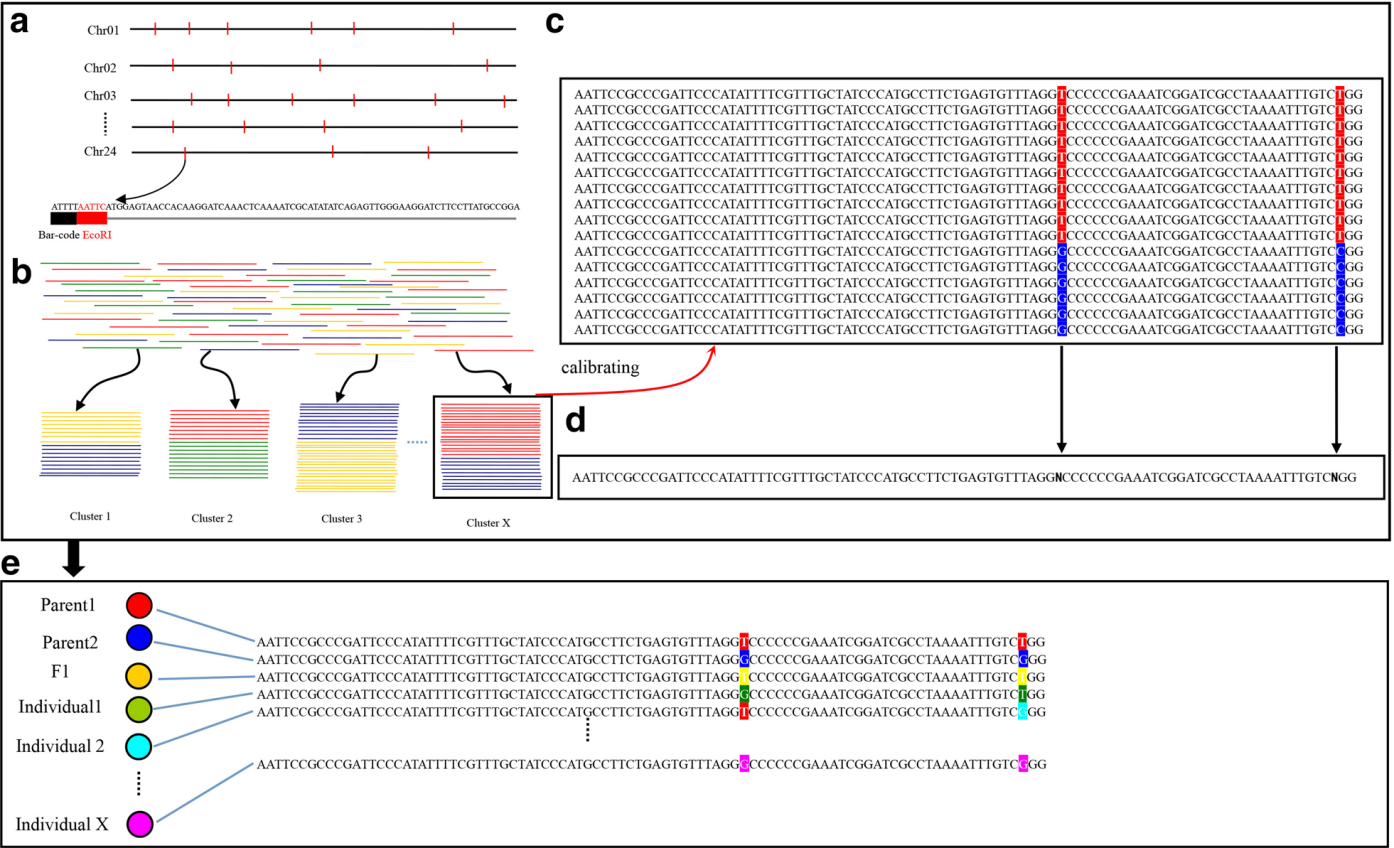
SNP calling based on DISR. **a** *Nicotiana tabacum* L. has 24 nuclear chromosomes, each of which contains multiple *Eco*RI cut sites (*red marks*). The genomic DNA is digested, bar coded with a population-specific sequence, and amplified resulting in multiple sequence reads from each of the RAD tag sites in the genome. Each sequence consists of a population-specific 5-bp barcode (*black*), the enzyme-recognition sequence (*red*), and the downstream sequence. **b** The de novo RAD tag pipeline compares all the sequenced reads and builds clusters of exactly matching tags. **c** Pair wise comparisons are made between all clusters. **d** There is a cluster in the locus that is SNP. **e** The consensus sequence for that RAD tag site within the population

Besides, the method based on reference, we have attempted to call SNPs by DISR. Instead, we used a multistep process to identify RAD tag loci within populations, assign a consensus sequence to each individual at each RAD tag locus, and align consensus sequences across populations (Fig. 5). A flowchart is also provided for clarity in Additional file 2.

Within each individual, identical reads were aligned together into clusters (other study termed it as stacks) (Fig. 5b–d). The pairwise sequence divergence among clusters was used to group them into putative loci (Fig. 5e). Loci were defined as a set of clusters such that for each cluster there is another cluster in the locus that is at most one nucleotide divergent. Clusters containing excessive numbers of sequence reads can occur when multiple, repetitive sites in the genome are all within a single nucleotide of one another. For this analysis, all clusters with a depth of coverage greater than two standard deviations above the mean cluster depth were removed and the remaining clusters were merged into a locus. For each nucleotide site in a locus, a likelihood ratio test of the read counts of alternative nucleotides was used to test whether the allele frequency of the most observed nucleotide was significantly larger than a threshold *p* following the method of Emerson et al. [43]. After these processes, an in-house perl script was used to integrate the clusters of parents and F_1_ progeny into a catalog and create a set of all possible loci in a mapping cross. Then, clusters of BC_1_ progenies are matched against the catalog to determine the genotype at each locus in every individual in the cross population.

### Genotyping and linkage mapping

Distorted markers (*p* < 0.01) were filtered off to construct a genetic map by a Chi square test (*χ*^2^ < 15 was selected for JoinMap 4.0) [35]. LGs were identified with an independent logarithm of odds (LOD) threshold of 7. Due to the large number of markers segregating in the population, if the number of the linkage group is more than 220, we used (in JoinMap 4.0) a maximum likelihood algorithm mapping the marker order for calculation efficiency [44]. We also calculated genetic distances (cM) using Haldane’s mapping function. However, the scope of corresponding linkage groups (3000–6000 cM) exceeded JoinMap 4.0 and therefore, the linkage length was divided by 100 for map presentation. In other linkage groups whose maker number was equal or less than 220, a linear regression algorithm and Kosambi’s mapping function was used for map construction and genetic distance estimation [45]. Following the initial mapping, potential errors that appeared as doubtful double-recombinants were identified using genotype probabilities function of JoinMap 4.0 [35] (*p* < 0.001). The suspicious genotype was replaced by a missing value as suggested by Isidore et al. [46] and Van Ooijen [35]. A linkage map was then constructed afresh using the corrected dataset. Potential error elimination and linkage map construction was iterated until no dubious genotype was identified. Markers with >45 % missing values or distorted (*χ*^2^ test, *p* < 0.001, d.f. = 2) were removed in each step of the iteration.

## Additional files

**Additional file 1.** Library detail information.
**Additional file 2.** A flowchart for bioinformatic analysis procedure in this study.
