# Supplementary figures and images for: SNP-based genetic linkage map of tobacco (Nicotiana tabacum L.) using next-generation RAD sequencing
Source: J Biol Res (Thessalon). 2015 Oct 6;22:11. doi: 10.1186/s40709-015-0034-3 (PMC4607152; doi:10.1186/s40709-015-0034-3)

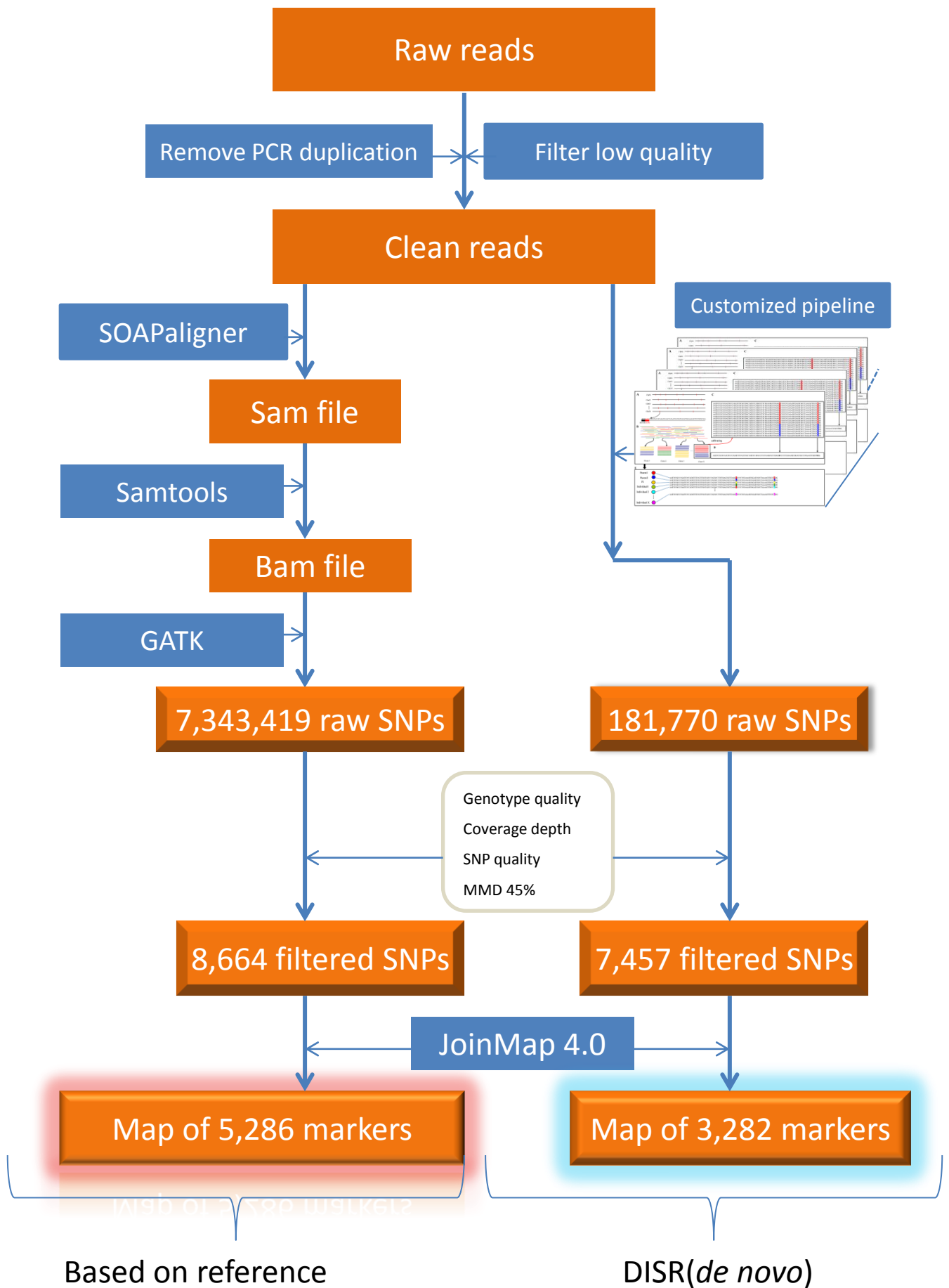

Supplement: Supplementary file 2 — Additional file 2. A flowchart for bioinformatic analysis procedure in this study. [file 40709_2015_34_MOESM2_ESM.pdf]
